# An in vitro micro-computed tomography analysis of internal voids: single-shade bulk-fill versus high-strength restorative composite

**DOI:** 10.1007/s10266-026-01393-5

**Published:** 2026-04-15

**Authors:** Merve Ağaccıoğlu, Merve Nur Yılmaz, Ali Keleş

**Affiliations:** 1 Department of Restorative Dentistry, Faculty of Dentistry, Bolu Abant Izzet Baysal University, Bolu, Turkey; 2 Department of Endodontics, Faculty of Dentistry, Bolu Abant Izzet Baysal University, Bolu, Turkey

**Keywords:** Single-shade, Bulk-fill, Micro-CT, Void, Porosity

## Abstract

This study aimed to investigate the internal void formation of two single-shade bulk-fill flowable composite resin and a high-strength restorative composite at different thickness levels by 3D micro-computed tomography (CT) analysis. Two different single-shade flowable bulk-fill composite resins (Charisma Bulk Flow One-CH and Omnichroma Flow Bulk-OM), and a high-strength restorative composite (G-aenial Universal Injectable-INJ) were used in this study. Cylinder-shaped samples were prepared at three different thicknesses (2, 4, and 6 mm) from each test material (n = 3). Samples were scanned utilising a micro-CT device, and internal voids of the materials were evaluated. Data were analyzed with Jamovi v2.7.6 and Mi nitab v14. Robust ANOVA was applied when normality was not met for the interaction between material and thickness. Multiple comparisons were analyzed with Holm-corrected robust t-test. Generalized linear models were used when normal distribution was met. Multiple comparisons were employed with Tukey test. Significance level was set as *p* < 0.05. Material*thickness interaction did not significantly affect total porosity percentages of the tested groups (*p* > 0.05), whereas it influenced total number of voids significantly (*p* < 0.001). Regardless of different thicknesses, the composite resin’s median total porosity and the number of total void values from the lowest to the highest total porosity percentages were as follows: INJ < OM < CH. The total number of voids increased as the thickness increased for the bulk-fill composite resins. Single-shade bulk-fill composite resins exhibited higher total porosity values.

## Introduction

Composite resins are the most frequently used materials for both anterior and posterior restorations, due to patient demand and ongoing material improvements [1–3]. Bulk-fill composite resins were introduced to provide an easier composite resin placement process for clinicians by eliminating the need for 2 mm increments and reducing chair-time. These composite resins can be used in increments of 4 mm or higher, as specified by the manufacturer's instructions. Moreover, some bulk-fill composite resins require an outer layer of a conventional composite resin, known as a ‘capping layer’. There are both packable (high-viscosity) and flowable (low-viscosity) bulk-fill composite resins available in the market. These materials achieve a higher depth of cure by their various properties and compositions [4]. Increased thickness of a single composite resin increment may influence its degree of conversion, polymerization shrinkage, and mechanical properties [5, 6], and also may cause insufficient marginal adaptation and internal voids.

The bulk-fill composite resins that are included in this study are Charisma Bulk Flow One (CH) and Omnichroma Flow Bulk (OM). OM is a single-shade flowable bulk-fill composite resin with smart chromatic technology and does not require a capping layer [7]. There are various thicknesses recommended when polymerized with different light-intensity options, i.e., 4.2 mm thickness with 1200 mW/cm^2^ for 30 s [8]. Charisma Bulk Flow One is a single-shade flowable bulk-fill composite resin with adaptive light matching, which does not require a capping layer. It is recommended to place a 4 mm thickness for a single increment with 600–1550 W/cm^2^ for 20 s [9]. There are few studies regarding the color stability, surface roughness, or other mechanical properties of these materials [12–14]. However, there are yet no studies that investigate their internal void formation. An injectable restorative composite resin, G-aenial Universal Injectable (INJ), was also used in this study. It is recommended that a 2.5 mm thickness of G-aenial Universal Injectable be polymerized for 10 s with > 1200 mW/mm^2^ for A1, A2, A3, B1, B2, JE, and AE shades [10]. For darker and more opaque shades of the material, it is advised to be applied in smaller increments (≤ 2 mm). G-aenial Universal Injectable can be used by both conventional layering and injection molding techniques in various clinical cases. The injection molding technique, which includes a replicated diagnostic wax-up by a flowable composite resin applied with a transparent silicone index, is another recent development in restorative dentistry [11]. There is yet a single study investigating the internal voids of G-aenial Universal Injectable [12]. The more detailed layering instructions of composite resins highlight the demanding technical sensitivity of employing such materials for a broader range of clinical cases.

Micro-computed tomography (CT) has a wide range of applications in dental analysis, from high-density materials such as ceramics and metal implants to soft tissues infused with contrast agents. In dental materials, closed internal voids that have no relation to the outer surface of the material are imperfections that may disrupt their mechanical properties, resulting in higher water absorption, leading to clinical failures. Internal voids can be quantified with micro-CT imaging and analysis [13–17]. It was reported that application tip diameter, viscosity of the material, irregularities between layers, and air bubbles during the manufacturing and packaging processes may be the reasons for internal void formation [18]. There are studies that evaluated different designs of increments regarding marginal adaptation and void formation of bulk-fill composite resins [18, 19]. Thus, novel single-shade flowable bulk-fill composite resins and different total thicknesses (2, 4, and 6 mm), with appropriate increments according to the manufacturers’ instructions, were included in this study.

It is essential for clinicians to be aware of the most effective layering approaches, considering reduced chair-time for wide cavities without jeopardizing the restoration’s mechanical properties. This study aimed to investigate the internal void formation of two single-shade bulk-fill flowable composite resins and a high-strength restorative composite at different thickness levels using 3D micro-CT analysis. The null hypotheses of the study were as follows: (1) there would be no significant differences in total porosity percentages within the same composite resin groups of different thicknesses, (2) there would be no significant differences in total porosity percentages within the same thickness groups of the tested composite resins.

## Materials and methods

### Sample preparation

**Fig. 1:**
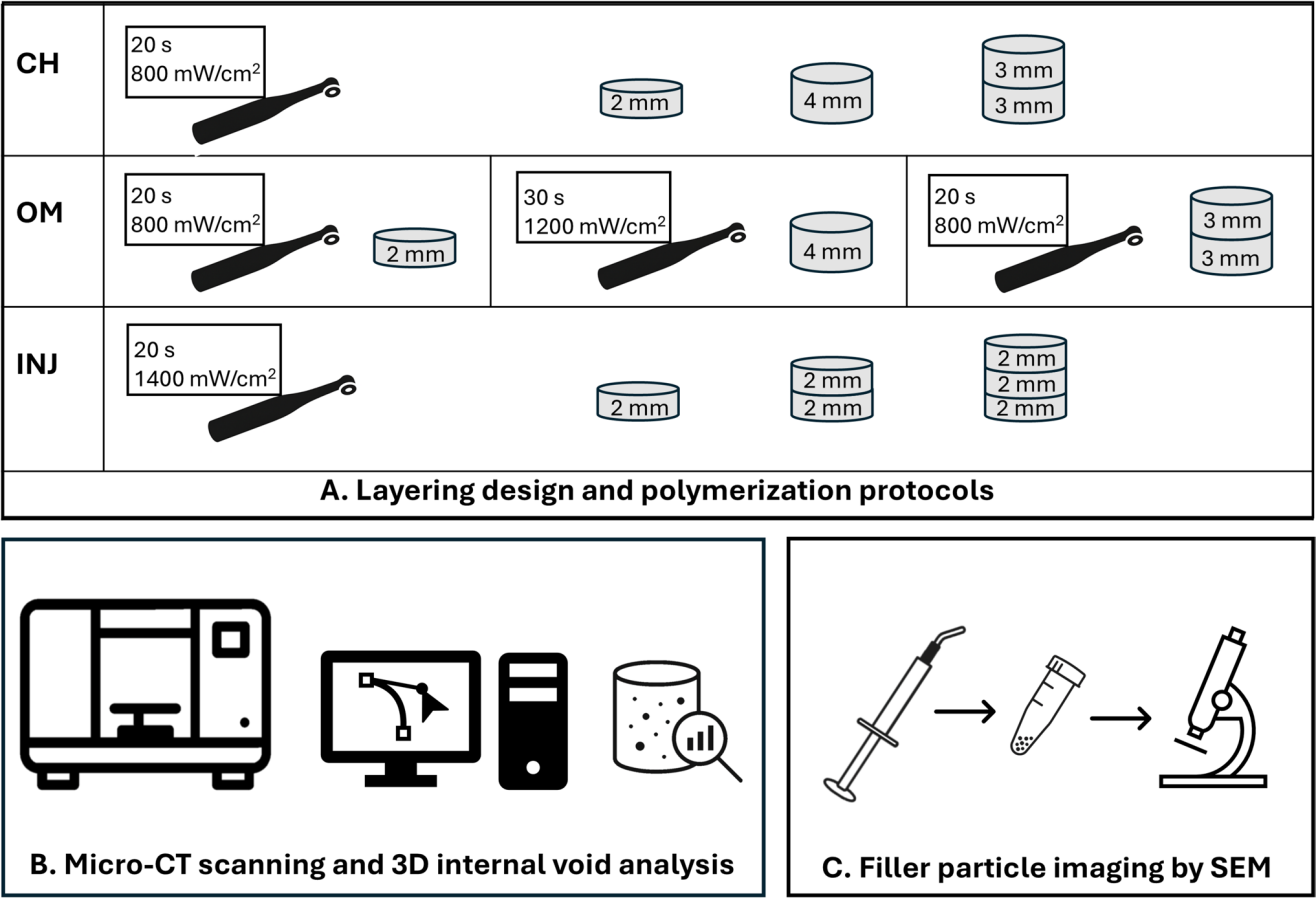
The study workflow illustration. **A** Polymerization protocols and layering design, **B** Micro-CT scanning and 3D internal void analysis, and **C** Filler particle imaging by SEM

Two different single-shade flowable bulk-fill composite resins (Charisma Bulk Flow One (Kulzer, Germany) and Omnichroma Flow Bulk (Tokuyama, Japan)), and a high-strength restorative composite (G-aenial Universal Injectable (GC Corp., Japan) in A2 shade) were used in this study. The compositions and properties of the tested materials are presented in Table 1. A two-piece adjustable Teflon mold was used to create cylinder-shaped (4 mm in diameter) composite resin samples, prepared with different thicknesses (2, 4, and 6 mm) for each test material. All of the tested materials were placed as a single layer for 2 mm thickness groups. The INJ was layered in two increments, whereas bulk-fill materials were placed as a single increment for the 4 mm thickness groups. Three 2 mm-layers were placed for INJ, and two 3 mm-layers were incremented for the bulk-fill composite resins for the 6 mm thickness groups. The composite resins were polymerized with an LED curing unit (VEGA LED, Öncü Dental, Türkiye). Layer design of the thickness groups and the polymerization protocols can be seen in Table 2 and Fig. 1. The sample size for this study was calculated as n = 3 with 0.95 of power and 0.05 of error level (G.Power-3.1.9.2 software) [20, 21]. A total of 27 specimens were prepared, creating 9 test groups.

**Table 1:** Product list and properties of the tested materials

Material	Matrix	Filler	Filler rate (%wt/vol)	Lot no
Charisma bulk flow one	Bis-EMA, UDMA, and TEGDMA	0.02 μm-5 μm, Ba-AI-F silicate glass, YbF_3_ and SiO_2_	65/41	M010024
Omnichroma flow bulk	UDMA and TEGDMA	0.2–0.4 µm, spherical silica-zirconia fillers	69/55	0241
G-aenial universal injectable	Bis-EMA, TCD-DMA, methacrylate monomers (NPGDMA and melamine formaldehyde resin), and UDMA	0.15 µm glass-filler (barium glass), silica	69/–	2210141

*Bis-EMA* Bisphenol A ethoxylated dimethacrylate, *UDMA* Urethane dimethacrylate, *TEGDMA* Triethylene glycol dimethacrylate, NPGMA: 2,2-dimethyl-1,3-propanediyl bismethacrylate

**Table 2:** Layer design of the different thickness groups for the tested materials

Material	Thickness groups		
	2 mm	4 mm	6 mm
Charisma bulk flow one	Single increment 20 s–800 mW/cm^2^	Single increment 20 s–800 mW/cm^2^	Two 3 mm-increments 20 s–800 mW/cm^2^
Omnichroma flow bulk	Single increment 20 s–800 mW/cm^2^	Single increment 30 s–1200 mW/cm^2^	Two 3 mm-increments 20 s–800 mW/cm^2^
G-aenial universal injectable	Single increment 10 s–1400 mW/cm^2^	Two 2 mm-increments 10 s–1400 mW/cm^2^	Three 2 mm-increments 10 s–1400 mW/cm^2^

### Micro-CT analysis

Each sample was placed on the rotating platform of a micro-CT device (Skyscan 1172; Bruker Micro-CT, Kontich, Belgium) with 0.5-mm-thick aluminium and 0.38-mm-thick copper filters at 85 kV, 118 µA, and a 1368 µm pixel size. The scanning procedure was performed with a 180° rotation around the vertical axis, a 200 ms camera exposure time, a 0.7° rotation step, 2 frame averaging, and 20 random movements. Each scan lasted approximately 40 min.

The raw data were reconstructed using NRecon software (NRecon v.1.7.1.1, Bruker Micro-CT) with a 30% beam hardening correction, ring artifact correction of 7, and smoothing of 2. CTAn (version 1.18.8, Bruker Micro-CT) was employed to analyze the data. The volume of interest (VOI) was selected as the part extending from the bottom surface of the material up to the top of the material. 3D analysis was used to determine the material volume, total void volume, the number of large and small voids, and the volume of large and small voids in VOI (a small void was set as < 40 voxels (approximately < 0.01 mm^3^)). The total number of voids was calculated as the sum of large and small voids.

The total porosity percentage (%), large void volume percentage (%), and the number of large void percentage (%) values were calculated:

$$Total\  Porosity\  Percentage = \frac{Total\ Void\ Volume \times 100}{Total\ Composite\ resin\ Volume + Total\ Void\ Volume}$$

$$Large\ Void\ Volume\ Percentage = \frac{Large\ Void\ Volume \times 100}{Total\ Void\ Volume}$$

$$Number\ of\ Large\ Void\ Percentage = \frac{Number\ of\ Large\ Voids \times 100}{Number\ of\ Total\ Voids}$$

CTVol (Bruker Micro-CT, v. 2.3.2) was employed for 3D visualization of a representative sample.

### Scanning electron microscopy (SEM) analysis

Each composite resin underwent SEM analysis (Jeol JSM-7001F, Tokyo, Japan; 15 kV accelerating voltage) for filler particle visualization. Organic matrix structures were dissolved by storing the composite resins in acetone before imaging. The images were acquired at a magnification of × 10,000.

### Statistical method

Data were analyzed employing Jamovi v2.7.6 and Minitab v14. The Shapiro–Wilk test was used to examine the assumption of normality. Robust ANOVA was utilized to compare the data when the normal distribution was not met for the interaction between material and thickness. Multiple comparisons were analyzed with Holm-corrected robust t-test. Generalized linear models were used for the comparisons when normal distribution was met. Multiple comparisons were carried out with Tukey test. Quantitative data were presented as median (min- max) and mean ± standard deviation. Significance level was determined as *p* < 0.05.

## Results

**Fig. 2:**
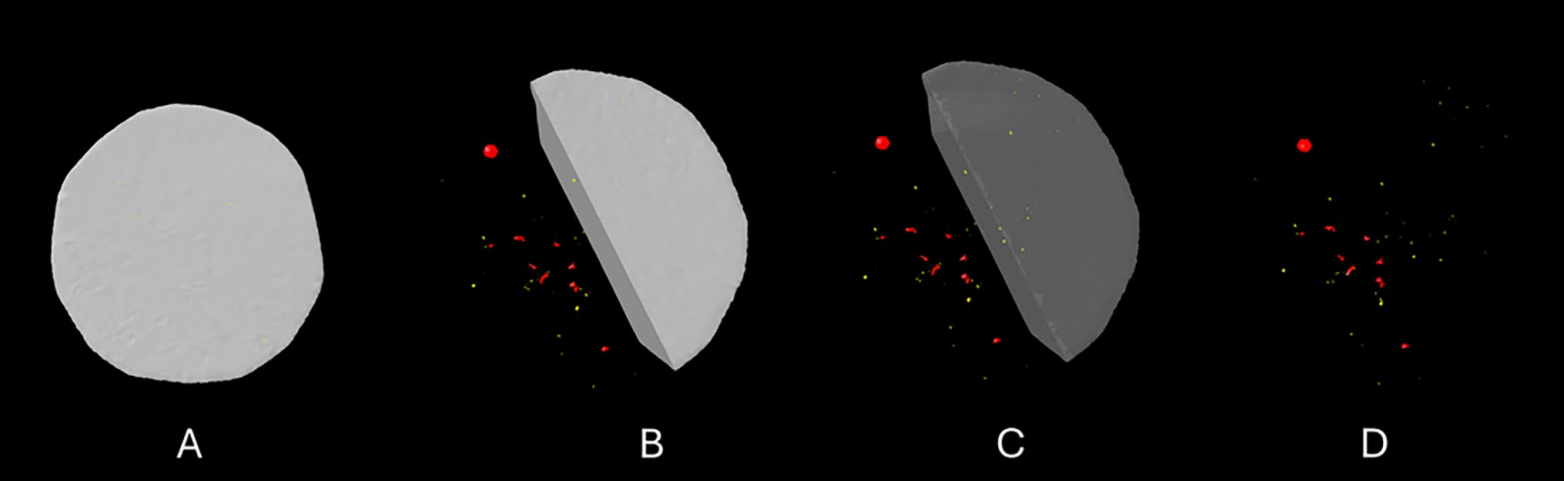
A 3D model* of a representative sample. **A** Top view of the sample. **B** Cut-away view of the composite resin with the visible internal voids. **C** Translucent half view of the composite resin with visible internal voids. **D** Internal large and small voids. *Composite resin: Gray, Large voids: Red, Small voids: Yellow

**Fig. 3:**
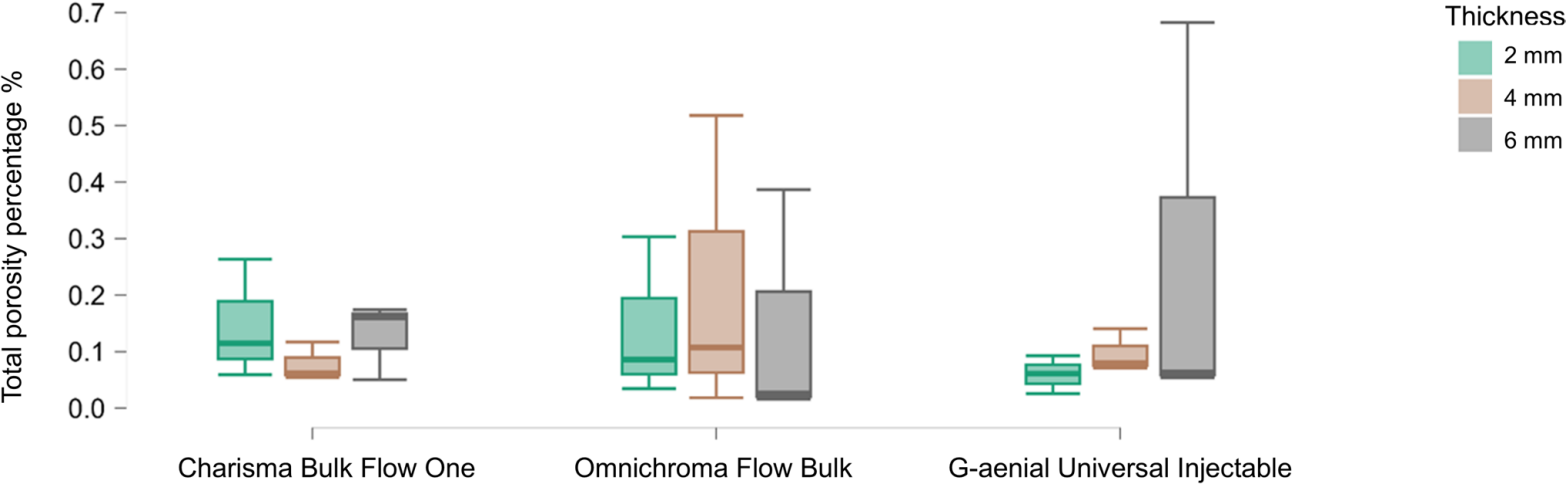
Box-plot graph of the total porosity percentage (%) values

**Fig. 4:**
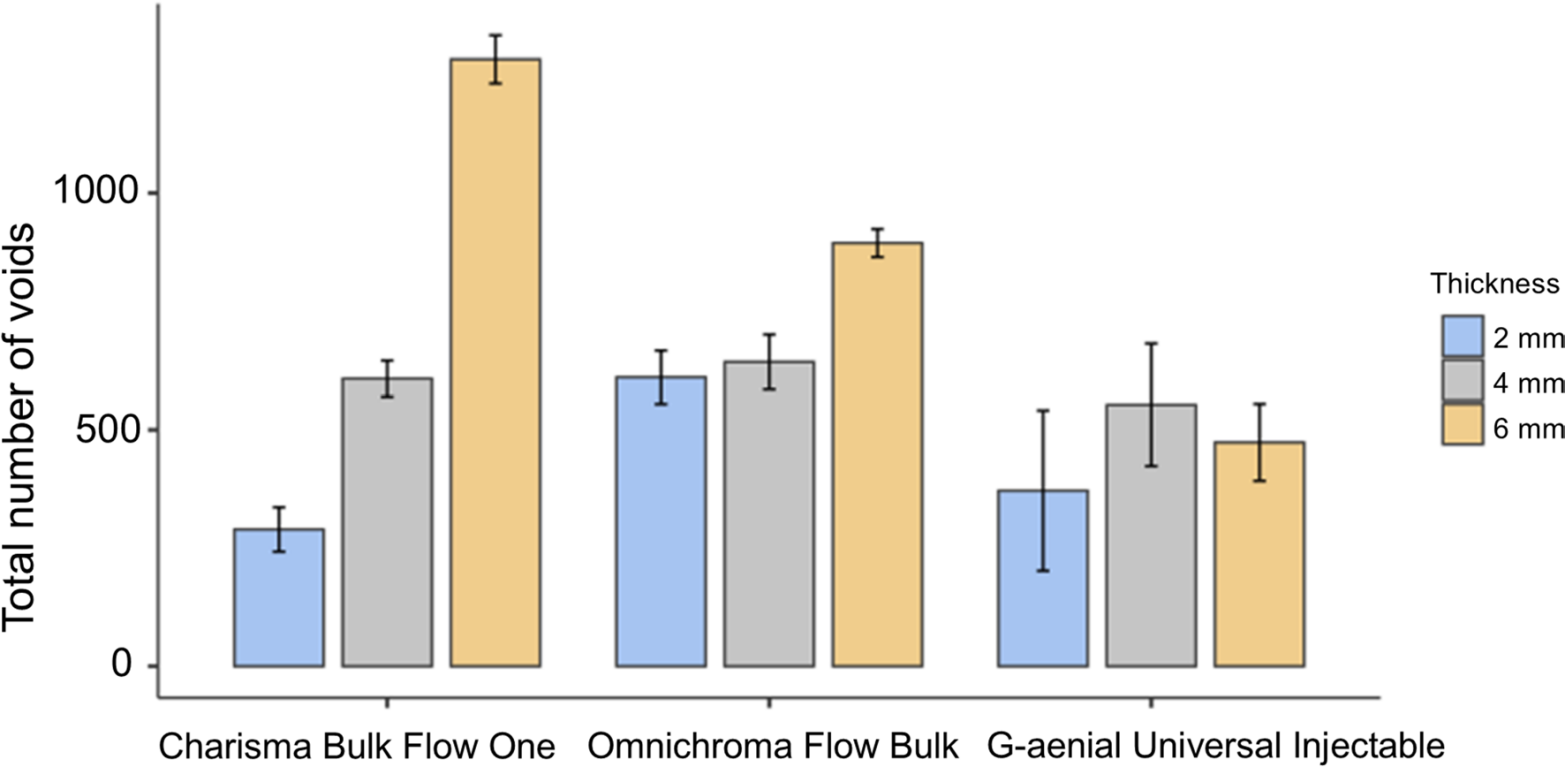
Bar graph of the total number of voids

A 3D model visual of a representative sample can be seen in Fig. 2. Total porosity percentage, the number of total voids, the large void volume percentage, and the number of large void percentage results are seen in Tables 3, 4, 5, and 6, respectively. Box plot and bar graphs of total porosity percentage and the number of total voids, respectively, can also be seen in Figs. 3 and 4. Material and thickness main effects and material*thickness interaction did not affect total porosity percentages significantly (*p* = 0.509, *p* = 0.994, and *p* = 0.113, respectively) (Table 3). Regardless of different thicknesses, the composite resin’s median total porosity values from the lowest to the highest total porosity percentages were as follows: INJ < OM < CH (Table 3). Overall, the lowest and the highest total porosity percentages were observed in OM-6 mm (0.026 (0.015–0.387)) and CH-6 mm (0.16 (0.05–0.174)) groups, respectively (Table 3).

**Table 3:** Total porosity percentage (%) comparisons based on different material and thickness groups

Material	Thickness			Total		Test statistics	*p*^*x*^
	2 mm	4 mm	6 mm				
Charisma bulk flow one	0.115 (0.059–0.263)	0.062 (0.054–0.117)	0.16 (0.05–0.174)	0.115 (0.05–0.263)	Material	0.676	0.509
Omnichroma flow bulk	0.086 (0.034–0.303)	0.107 (0.018–0.518)	0.026 (0.015–0.387)	0.086 (0.015–0.518)	Thickness	0.006	0.994
G-aenial universal injectable	0.061 (0.026–0.093)	0.079 (0.07–0.141)	0.063 (0.053–0.682)	0.07 (0.026–0.682)	Material * thickness	7.477	0.113
Total	0.086 (0.026–0.303)	0.079 (0.018–0.518)	0.063 (0.015–0.682)				

^x^Robust ANOVA; median (min—max)

**Table 4:** The number of total voids comparisons based on different material and thickness groups

Material	Thickness			Total		Test statistics	*p*^x^
	2 mm	4 mm	6 mm				
Charisma bulk flow one	289.333 ± 81.206^C^	607.667 ± 66.154^BC^	1283 ± 88.357^A^	726.667 ± 444.739^a^	Material	8.956	**0.002**
Omnichroma flow bulk	610.667 ± 97.675^BC^	643 ± 100.18^BC^	894.333 ± 51.189^AB^	716 ± 153.734^a^	Thickness	22.091	** < 0.001**
G-aenial universal injectable	371.333 ± 293.5^C^	552.667 ± 224.11^BC^	473.333 ± 140.763^BC^	465.778 ± 212.704^b^	Material * thickness	8.801	** < 0.001**
Total	423.778 ± 215.585^a^	601.111 ± 133.092^a^	883.556 ± 361.308^b^	636.148 ± 311.678			

Significant differences (*p* < 0.05) are indicated in bold

^x^Generalized linear models; mean ± standard deviation

^a−b^The material groups with the same lowercase superscript letters signify no statistical difference

^a−b^The thickness groups with the same lowercase superscript letters signify no statistical difference

^A−C^Same capital superscript letters signify no statistical difference between the material*thickness interaction groups

**Table 5:** Large void volume percentage (%) comparisons based on different material and thickness groups

Material	Thickness			Total		Test statistics	*p*^x^
	2 mm	4 mm	6 mm				
Charisma bulk flow one	93.435 (92.941–96.698)^A^	77.057 (66.261–87.404)^B^	89.449 (59.526–89.458)^BC^	89.449 (59.526–96.698)	Material	2.910	0.054
Omnichroma flow bulk	73.542 (60.175–94.63)^BC^	89.225 (42.228–97.672)^ABC^	51.034 (25.086–97.2)^ABC^	73.542 (25.086–97.672)	Thickness	1.130	0.322
G-aenial universal ınjectable	86.505 (53.624–91.657)^BC^	91.378 (80.093–94.55)^ABC^	87.689 (82.675–99.499)^AC^	87.689 (53.624–99.499)	Material * thickness	20.170	** < 0.001**
Total	91.657 (53.624–96.698)	87.404 (42.228–97.672)	87.689 (25.086–99.499)	87.689 (25.086–99.499)			

Significant differences (*p* < 0.05) are indicated in bold

^x^Robust ANOVA; median (min—max)

^A−C^Same capital superscript letters signify no statistical difference between the material*thickness interaction groups

**Table 6:** The number of large void percentage (%) comparisons based on different material and thickness groups

Material	Thickness			Total		Test statistics	*p*^x^
	2 mm	4 mm	6 mm				
Charisma bulk flow one	4.445 ± 2.891	3.764 ± 0.824	4.886 ± 0.452	4.365 ± 1.597^b^	Material	8.835	**0.002**
Omnichroma flow bulk	4.765 ± 1.63	5.015 ± 3.135	4.358 ± 2.579	4.713 ± 2.206^b^	Thickness	0.211	0.812
G-aenial universal injectable	10.461 ± 4.983	8.378 ± 4.199	10.111 ± 3.047	9.65 ± 3.724^a^	Material * thickness	0.177	0.948
Total	6.557 ± 4.19	5.719 ± 3.362	6.452 ± 3.409	6.243 ± 3.549			

Significant differences (*p* < 0.05) are indicated in bold

^x^Generalized linear models; mean ± standard deviation

^a−b^Same lowercase superscript letters signify no statistical difference

Both material and thickness main effects and also material*thickness interaction significantly affected the number of total voids results (*p* = 0.002, *p* < 0.001, and *p* < 0.001, respectively) (Table 4). Regardless of different thickness groups, the overall number of total voids from the lowest to the highest was as follows: INJ < OM < CH (Table 4). CH-6 mm had a significantly higher number of total voids than all of the other test groups (*p* < 0.05) except for OM-6 mm (*p* > 0.05) (Table 4). CH-2 mm had a significantly lower number of total voids than only the CH-6 mm and OM-6 mm groups (*p* < 0.05) (Table 4).

Material*thickness interaction significantly affected (*p* < 0.001) the large void volume percentages of the test groups, whereas material and thickness main effects did not (*p* = 0.054 and *p* = 0.322, respectively) (Table 5). The highest and the lowest median large void volume percentage was observed in CH-2 mm (93.435 (92.941–96.698)) and OM-6 mm (51.034 (25.086–97.2)) groups, respectively (Table 5). There were no statistical differences between different thickness groups of the same composite resins (*p* > 0.05) except for CH regarding large void volume percentages (Table 5). CH-2 mm exhibited significantly higher large void volume percentage, whereas there was no statistical difference between CH-4 mm and CH-6 mm (*p* > 0.05) (Table 5).

Only the material type, as a main effect, significantly affected the number of large void percentage (*p* = 0.002) (Table 6). INJ had significantly higher mean total number of large void percentage (9.65 ± 3.724) than CH (4.365 ± 1.597) and OM (4.713 ± 2.206) (*p* < 0.05) (Table 6). There was no statistical difference between CH and OM’s total number of large void percentage (*p* > 0.05) (Table 6).

**Fig. 5:**
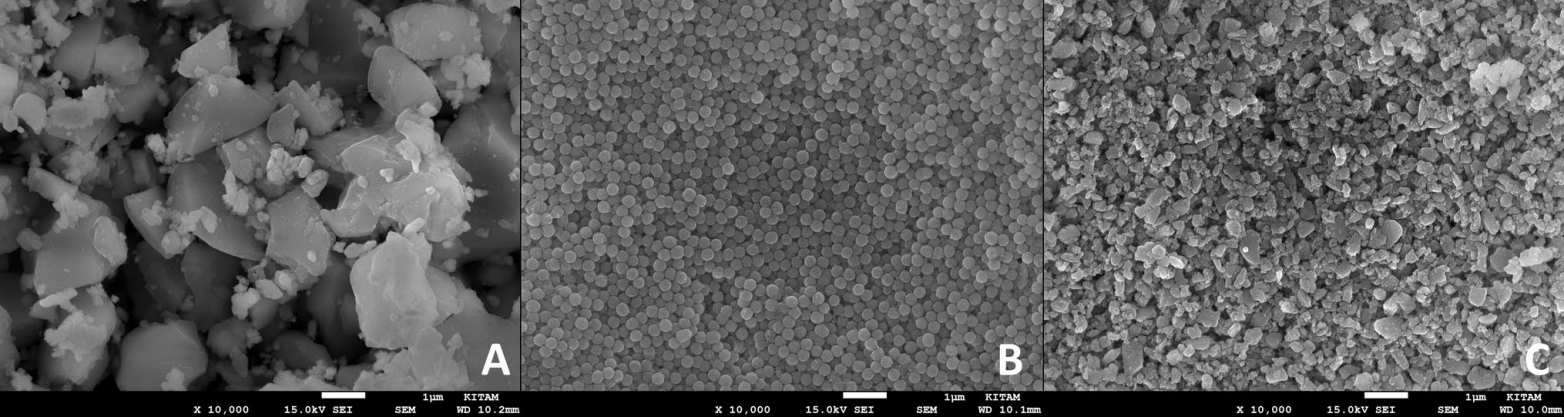
SEM images of the filler particles of the composite resins at × 10000 magnification. **A** Charisma bulk flow one, **B** Omnichroma flow bulk, **C** G-aenial universal injectable

SEM images of filler particles of the composite resins are presented in Fig. 5. Nanohybrid and nanofill structures of the composite resins can be clearly identified by the size of the fillers.

## Discussion

In this study, the internal void formation of two single-shade bulk-fill flowable composite resin and a high-strength restorative composite at different thickness levels was investigated by 3D micro-CT analysis. Thickness did not have a significant effect on total porosity percentages (*p* = 0.994) so there were no significant differences within the same composite resin groups of different thicknesses (Table 3). Thus, the first null hypothesis was accepted. Material was not found as statistically effective on total porosity percentages (*p* = 0.509) so there were no significant differences within the same thickness groups of the tested composite resins (Table 3). As a result, the second hypothesis was also accepted.

Micro-CT allows a 3D assessment of the material structure without an invasive procedure on the material sample. Thus, it is often used for porosity analysis of composite resin restorations in the literature [14, 15, 22]. The manual modelling necessity of composite resins [16] and also air bubbles due to the application process may cause voids within the materials. Closed voids may disrupt the mechanical integrity of the materials, leading to crack formation, followed by bulk failure [14]. Clinical indications recommended by the manufacturers are similar for the tested materials in this study. Unlike the other tested composite resins, class IV cavities are not listed among the clinical indications of CH. A study evaluated the porosity of different composite resins in their compule and restoration forms, and it was stated that the amount of porosity (vol%) values decreased in restoration forms of the composite resins [17]. Another previous study evaluated the void formation of the sonic delivery method with various composite resins. It was reported that sonic delivery might increase porosity depending on the composite resin type [14]. These results may suggest that the proper application and handling can reduce the risk of porosity in the final form of the materials.

Although material, thickness, and material*thickness interaction were not significantly effective on total porosity (Table 3), all of these main effects significantly affected the number of total voids (Table 4). Regardless of thickness, total porosity, and the number of total voids from the lowest to the highest were as follows: INJ < OM < CH (Tables 3 and 4). The lower results of INJ may be due to its 2 mm-increment placement, and it is also not a bulk-fill composite resin, unlike the other tested materials. In addition, it was claimed that INJ has a highly thixotropic viscosity property. Thus, this thixotropic structure allows the material not to slump during placement, but to flow when moved around with an instrument [10]. These qualities may be advantageous for decreasing the risk of air bubble occurrence as the material is extruded from the syringe’s tip. A micro-CT analysis study investigated 2.5 mm-thickness fifteen restorative materials, including INJ [12]. It was reported that INJ demonstrated a lower porosity percentage than the flowable bulk-fill composite resins (SDR flow + and Filtek Bulk Fill Flowable), but there were no statistical differences between these materials (*p* > 0.05). Total porosity values of INJ (0.076 ± 0.018) were similar to our results of total porosity of INJ (0.07 (0.026–0.682)) (Table 3).

In clinical practice, commonly treated class II restorations require restorative material of 4–5 mm thickness [18]. Even though the composite resins have acceptable mechanical properties, internal voids of the restorations may jeopardise the materials’ performance under loading [13]. The number of total voids, regardless of composite resins, in the total thickness groups was observed from the highest to the lowest as follows: 6 mm > 4 mm > 2 mm (Table 4). This result may be due to the total material volume increase. Incremental layering of thin composite resins reduces air entrapment. While flowable bulk-fill composite resins still carry an air entrapment risk during injection, they eliminate the need for multiple layers. This prevents the voids that typically form when adapting and manipulating traditional composite resins with hand instruments.

Although internal voids are considered defects, an acceptable amount of porosity or number of voids for the clinical performance of the materials has not yet been reported. The total porosity of the tested materials in the current study were observed as 0.026–0.115% (Table 3) and the number of large void percentages ranged in 3.764–10.111% (Table 6). These results highlight that the overwhelming majority of the voids were small, which may reduce the risk of critical failures. Voids may act as initiation points or stress concentrators for crack and fracture propagation. Higher amounts of voids can also increase water sorption and subsequent discoloration [22].

There are various monomers in the organic matrix of the resin composites in the market, such as Bis-GMA, UDMA, TEGDMA, and Bis-EMA. The organic matrix composition can influence the composite resins’ properties regarding polymerization and, as a result, their mechanical properties [2]. A previous study reported that UDMA groups demonstrated higher depth of cure than the Bis-EMA groups [1]. All the tested materials in this study contain UDMA. INJ had the least UDMA ratio (0.5– < 1%) in its matrix, followed by CH (≥ 10– < 20%) and OM (10–30%). INJ had the most varied monomer composition (Bis-EMA;10– < 25%, TCD-DMA; 5– < 10%, NPGDMA; 25– < 5%, and melamine formaldehyde resin; 1– < 25%), which may provide the material with diverse properties. Even though the resin matrices of CH and INJ present differences, Bis-EMA ratios of the materials (≥ 10–25% and 10– < 25%, respectively) are similar. Bis-EMA is the ethoxylated analog of Bis-GMA. Bis-EMA differs in that it lacks two hydroxyl groups [2]. TEGDMA was reported to present higher polymerization shrinkage [23]. There are different ratios of TEGDMA in OM and CH, which may be attributed to their higher number of total voids than INJ (Table 4).

Filler particle properties may influence the microstructure of resin composites. It was argued that resin composites with a hybrid filler composition may reduce the risk of air entrapment, thereby improving the packing density of the material's inorganic structure [3]. On the other hand, OM, a nanofill composite resin with homogeneous spherical fillers (Fig. 5), performed better than CH in terms of total porosity and the number of voids. However, OM and CH are both single-shade materials; OM uniquely does not contain any dyes or pigments and achieves its single-shade property through structural color technology [7], whereas CH employs adaptive light matching technology. Further, CH, a nanohybrid resin composite, has irregularly shaped fillers (Fig. 5) with a filler size range of 0.02 μm to 5 μm, which may have affected its internal void formation. The nanofill INJ exhibited lower total porosity and number of total voids than nanohybrid CH (Tables 3 and 4). This may be due to the Full-Coverage Silane Coating (FSC) technology of INJ. FSC technology provides an almost fully coated filler surface with the silane coupling agent. INJ was reported to exhibit the lowest water sorption and the highest flexural strength compared to another injectable restorative and traditional flowable composite resin [24, 25]. The material, when injected, keeps its shape without slumping, providing improved thixotropy and reduced extrusion pressure [10]. The smaller filler size (0.15 µm) (irregularly shaped, Fig. 5) of INJ may contribute to its better results in total porosity (Table 3) and number of total voids (Table 4) than OM (0.2–0.4 µm).

In this study, the polymerization protocols for sample preparation including light intensity and irradiation time adhered strictly to each manufacturers’ instructions. Although variations in polymerization protocols could be viewed as a limitation, following these guidelines ensures the study mimics clinical reality. Particularly, composite resins such as single-shade bulk-fill and high-strength restorative require unique irradiation parameters tailored to their specific layer thicknesses.

Micro-CT analyses for void formation also have some limitations, such as long scanning times, complex data processing, and limited device capacity for multiple-sample analysis compared to 2D digital radiography [26]. As a limitation of the study, the long-term effects of the oral environment, such as water sorption and thermal changes, were not simulated. These factors may impact the formation of voids and also the clinical consequences of materials’ porosity. The small sample size is another limitation of the study. Closed voids were investigated in the present study, it can be suggested that further studies may focus on the marginal adaptation of these materials in various cavity designs. In addition, employing mechanical analyses such as degree of conversion and microhardness would enrich the study and provide a more plausible interpretation of the clinical implications of porosity.

Within the limitations of the study, the following conclusions were drawn:

- There were no significant differences between the test groups in terms of total porosity percentages.
- The total number of voids increased as the thickness increased for the bulk-fill composite resins.
- Overall number of total voids of the composite resins from the lowest to the highest were as follows: INJ < OM < CH.

## Data Availability

The data generated in this study are included in this article.
